# Treatment of hereditary hemorrhagic telangiectasias with sclerotherapy: A case series

**DOI:** 10.1016/j.amjoto.2024.104537

**Published:** 2024-12-05

**Authors:** Jenny Ji, Andrew M. Peterson, Jay F. Piccirillo

**Affiliations:** a Department of Otolaryngology – Head and Neck Surgery, Washington University School of Medicine, 660 S Euclid Ave, CB 8115, St. Louis, MO 63110, USA; b Clinical Outcomes Research Office, Department of Otolaryngology-Head and Neck Surgery, Washington University School of Medicine, 660 S Euclid Ave, CB 8115, St. Louis, MO 63110, USA

**Keywords:** Hereditary hemorrhagic telangiectasia, Sclerotherapy, Lip, Tongue

## Abstract

**Background:**

Hereditary hemorrhagic telangiectasia (HHT) is characterized by abnormal blood vessel formation. One treatment for HHT-related arteriovenous malformations (AVMs) is sclerotherapy, which collapses the blood vessels by irritating the endothelial lining.

**Methods:**

This case series describes two HHT patients undergoing in-office sodium tetradecyl sulfate sclerotherapy for non-nasal telangiectasias and AVMs. The first patient had AVMs on the red lip while the second patient had an AVM and several telangiectasias on the tongue.

**Results:**

Both patients’ AVMs required only one treatment visit and were no longer noticeable within a week. Neither of the patients had any side effects from treatment.

**Conclusion:**

Sclerotherapy was used to successfully treat HHT-related AVMs in-office with no scarring and is an option that should be considered by providers.

## 1. Introduction

Hereditary hemorrhagic telangiectasia (HHT) is an autosomal dominant condition characterized by the presence of telangiectasias or arteriovenous malformations (AVMs) throughout the mucosal and cutaneous surfaces of the body, as well as the liver, lungs, brain, and spine [1]. There are a variety of treatments, including beta-blockers [2], sclerotherapy [3], laser surgery [3], and surgical excision [1], and each has its benefits and risks.

Sclerotherapy involves injecting a sclerosing agent to obliterate abnormal vascular lesions. Preparation of the sclerosing setup can be seen at www.youtube.com/watch?v=86vKjidhc2s&feature=em-share_video_user. Sclerotherapy is effective at treatment for varicose veins, but its efficacy in treating cutaneous vascular lesions is less clear.

Herein we describe two cases of successful treatment of HHT-related AVMs using sclerotherapy.

## 2. Case 1

This patient initially presented at 44 years old with severe HHT-related epistaxis. She received sclerotherapy injections for her nasal telangiectasias and returned several times per year for treatment. She was placed on tranexamic acid and propranolol for HHT-related bleeding.

She presented with two lip arteriovenous malformations (AVMs) that had been bleeding more frequently. Exam showed a 0.5 cm AVM entirely on the lower red lip and a 0.3 cm AVM on the vermilion border of the lower lip.

Both her nasal and lip telangiectasias were sclerosed during that visit. A 1:4 mixture of 3% sodium tetradecyl sulfate and air was injected into the lesions with a 25-gauge needle. A total of 1.2 ccs was used for the nose and lip. Bleeding was controlled with pressure, and the patient tolerated the procedure well.

**Fig. 1.**
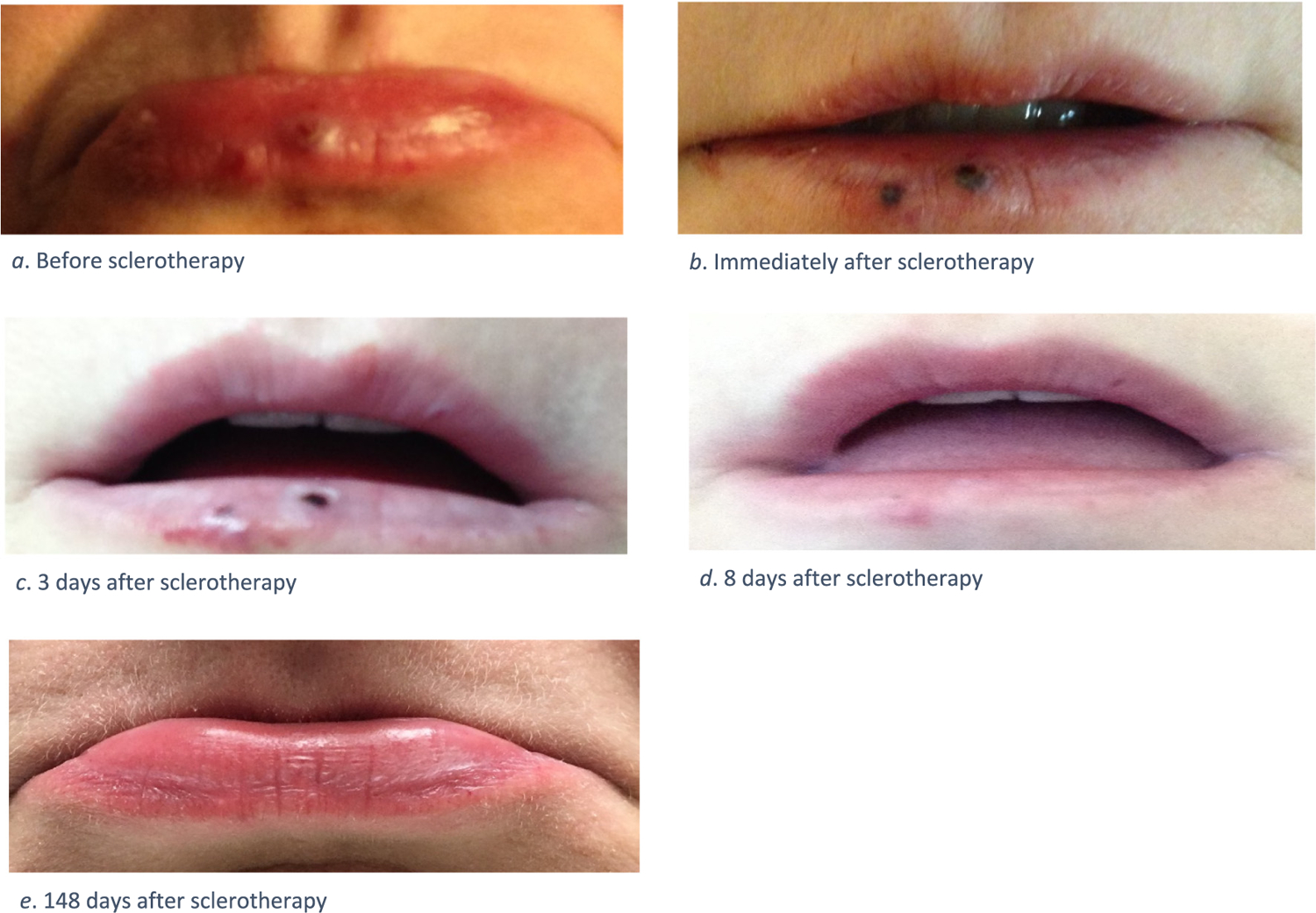
Photos of patient’s lip AVMs pre- and post-injection.

She returned to clinic around 5 months later and had no signs of telangiectasias or scarring on her lip. During the time between visits, she periodically took pictures of her lip to track her healing process, which demonstrated remarkable resolution of her AVMs without scarring (Fig. 1).

## 3. Case 2

This patient initially presented at 58 years old with persistent mouth, tongue, and nose bleeding and a history of multiple cauterization procedures to the nose and tongue. Over the next few years, she underwent an average of one sclerotherapy procedure a year for oral cavity telangiectasias. She used doxycycline for HHT-related bleeding and never required any sclerotherapy procedures for her epistaxis.

**Fig. 2.**
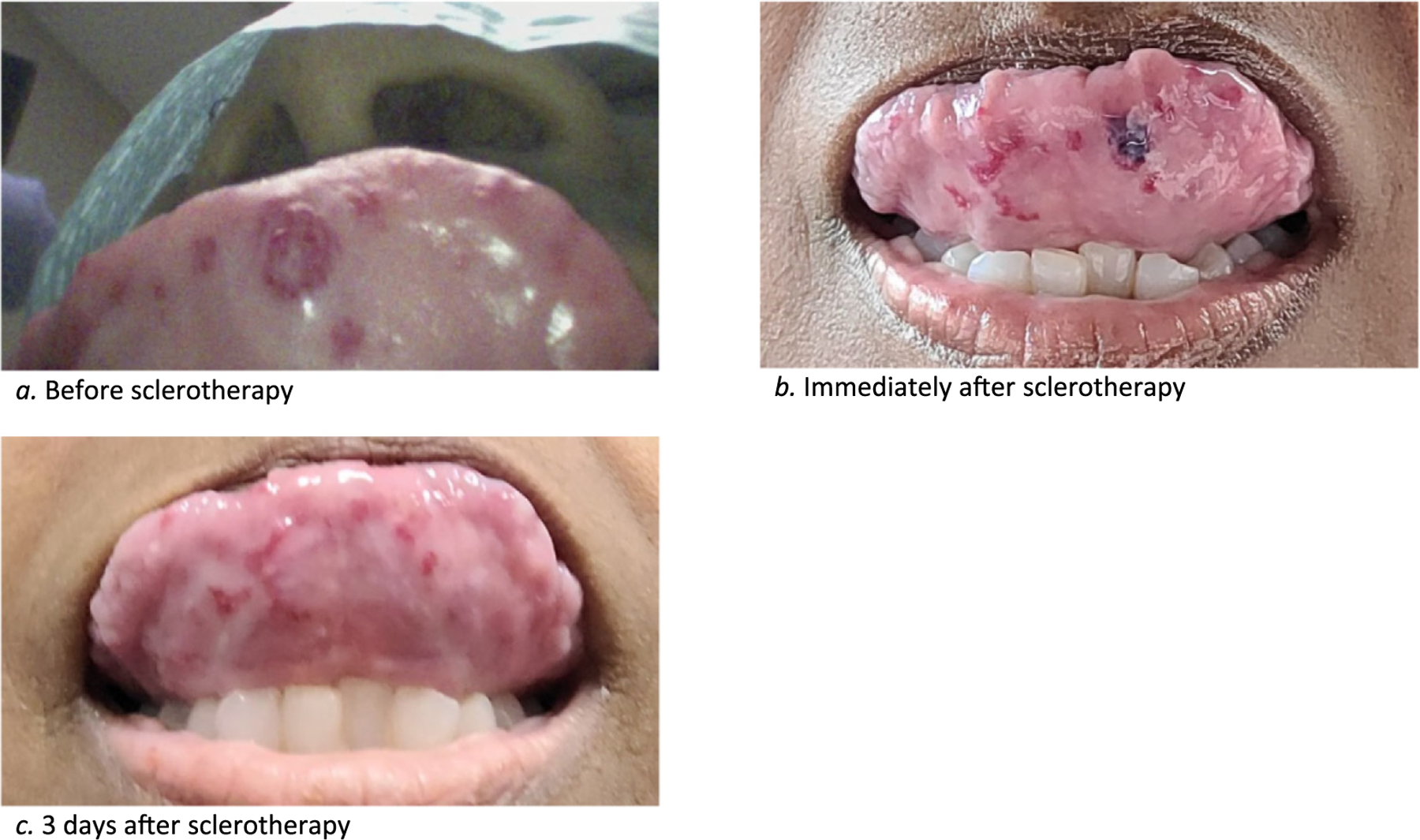
Photos of patient’s tongue AVM pre- and post-injection.

Most recently, she presented with multiple telangiectasias along the lateral tongue and one under the tip of her tongue. She stated that the AVM under the tip of her tongue was causing significant bleeding, so a 1:4 mixture of 3% sodium tetradecyl sulfate and air was injected into the lesions with a 25-gauge needle. Bleeding was controlled with pressure, and a second injection was performed a few minutes later. A total of 0.9 ccs was used for the tongue, and the patient tolerated the procedure well. Photos were taken before and after the procedure, demonstrating resolution of the treated lesion (Fig. 2).

## 4. Discussion

This report describes two cases of successful treatment of HHT-related AVMs with sclerotherapy. The first case involved two AVMs on the lip. This patient only required one treatment of sclerotherapy, and the lesion was barely noticeable after a week. The second involved an AVM under the tip of the tongue. The AVM was injected with sodium tetradecyl sulfate twice and the AVM was gone after three days.

Sclerosing agents like sodium tetradecyl sulfate irritate the endothelial lining of vessels, leading to the obliteration of the lumen through inflammation and thrombosis [4]. Sclerotherapy is a relatively non-invasive treatment for vascular lesions and is able to be safely administered in an outpatient clinic.

Traditional treatments of HHT-related vascular lesions involve cauterization, which is known to prompt vascular regrowth after damaging the tissue [5]. While lasers also have low rates of scarring, patients often require more visits to the office for treatment as compared to sclerotherapy [3].

While previous studies have mostly focused on nasal sclerotherapy, this case series provides two examples of successful treatment of non-nasal HHT-related AVMs and documents changes over time. Sclerotherapy is an option for the treatment of HHT-related cutaneous and mucosal vascular lesions.
